# Bioinformatic Analysis of Topoisomerase IIα Reveals Interdomain Interdependencies and Critical C-Terminal Domain Residues

**DOI:** 10.3390/ijms25115674

**Published:** 2024-05-23

**Authors:** Clark E. Endsley, Kori A. Moore, Thomas D. Townsley, Kirk K. Durston, Joseph E. Deweese

**Affiliations:** 1 Biological, Physical, and Human Sciences Department, Freed-Hardeman University, Henderson, TN 38340, USA; 2 FortyAU, Nashville, TN 37209, USA; 3 Department of Research and Publications, Digital Strategies, Langley, BC V2Y 1N5, Canada; 4 Department of Biochemistry, Vanderbilt University, Nashville, TN 37232, USA

**Keywords:** topoisomerase II, cancer, bioinformatics, intrinsically disordered domain, interdependency, DNA, topology, protein structure

## Abstract

DNA Topoisomerase IIα (Top2A) is a nuclear enzyme that is a cancer drug target, and there is interest in identifying novel sites on the enzyme to inhibit cancer cells more selectively and to reduce off-target toxicity. The C-terminal domain (CTD) is one potential target, but it is an intrinsically disordered domain, which prevents structural analysis. Therefore, we set out to analyze the sequence of Top2A from 105 species using bioinformatic analysis, including the PSICalc algorithm, Shannon entropy analysis, and other approaches. Our results demonstrate that large (10th-order) interdependent clusters are found including non-proximal positions across the major domains of Top2A. Further, CTD-specific clusters of the third, fourth, and fifth order, including positions that had been previously analyzed via mutation and biochemical assays, were identified. Some of these clusters coincided with positions that, when mutated, either increased or decreased relaxation activity. Finally, sites of low Shannon entropy (i.e., low variation in amino acids at a given site) were identified and mapped as key positions in the CTD. Included in the low-entropy sites are phosphorylation sites and charged positions. Together, these results help to build a clearer picture of the critical positions in the CTD and provide potential sites/regions for further analysis.

## 1. Introduction

Protein sequence analysis has been in use for decades and is an important tool in biochemistry to explore protein structure and function and clarify enzyme mechanisms [1,2,3,4,5]. One form of protein sequence analysis is to look for interdependencies within protein sequences using multiple sequence alignments [2,3,6]. Interdependencies can help identify positions and regions that may interact either proximally or by way of a long-distance relationships [2,6]. Previously, we reported the application of a derivation of the K modes clustering algorithm to the question of protein sequence interdependency using the PSICalc algorithm [6]. PSICalc identifies sites and site-clusters within a protein family sequence, represented by the columns in an MSA, that are, either structurally or functionally, mutually interdependent. It does this by creating a nested hierarchy of sites and clusters of sites determined by the level of mutual information they share within an MSA [2,6]. These associations enable the researcher to identify which site-interdependencies are critical for the function of a given protein family, be it structurally ordered or disordered. This study aims to utilize an updated version of PSICalc and other bioinformatic tools to make discoveries on a critical target of chemotherapy: topoisomerase II.

**Figure 1:**
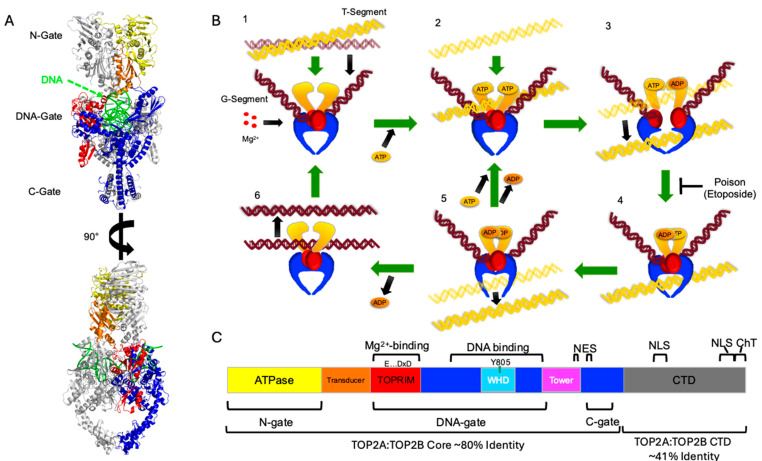
Domain structure and catalytic cycle of topoisomerase II. (**A**) Ribbon diagrams of the crystal structure of *S. cerevisiae* Top2 are shown as dimers rotated 90 degrees with one monomer colored and the other in gray. Color-coding matches part C. Gates are labeled at left. (**B**) The topoisomerase II catalytic cycle is shown in six stages, including (1) binding of gate-segment (G-segment); (2) binding of ATP and transport-segment (T-segment); (3) temporary cleavage of G-segment, opening of G-segment (DNA gate), hydrolysis of one ATP, transport of the T-segment; (4) closing of the G-segment, conformational change of the ATPase/transducer domains; (5) hydrolysis of the second ATP, opening of the C-gate and release of the T-segment; and (6) release of the ADP and of the G-segment. (**C**) Domain organization of topoisomerase II. Domains are labeled and color-coded. Winged-helix domain (WHD) within the DNA-binding region includes the active site tyrosine (Y805 in human Top2A). Nuclear export signals (NESs), nuclear localization signals (NLSs), and the Chromatin Tether domain (ChT) are also denoted. The C-terminal domain (CTD) is shown in grey and is not shown in parts A and B due to the unstructured nature of the region. Percent identity shown at the bottom based upon BLASTp comparison of P11388 Top2A with Q02880 Top2B sequences with CTD starting at 1175 (Top2A)/1193 (Top2B).

Humans express two type II topoisomerases, known as topoisomerase IIα and IIβ (Top2A and Top2B, respectively) [7,8,9]. These enzymes are found in the nuclei of all cells. Type II topoisomerases in eukaryotes are homodimers that unknot and alter the supercoiling state of DNA through a double-strand DNA cleavage and strand passage mechanism where the enzyme makes a temporary break in one double helix and passes another double helix through the break (Figure 1) [10,11]. Top2A expression fluctuates with the cell cycle and is most highly expressed in S-phase and M-phase consistent with its involvement in replication and mitosis [12,13,14,15,16]. Top2B is more involved in chromatin modulation and remodeling during transcription [12,13,14,16,17]. Type II topoisomerases have been the targets of anticancer drugs for decades because disruption of topoisomerase activity leads to DNA damage and consequently cell death [11,18]. Top2 inhibitors (e.g., dexrazoxane) block catalytic activity without increasing cleaved DNA while Top2 poisons (e.g., etoposide or doxorubicin) tend to lead to increased levels of DNA strand breaks [11,18]. 

Unfortunately, topoisomerase-targeted agents lack specificity, and both Top2A and Top2B are affected by the common Top2 inhibitors and poisons [11,19]. Most of the clinically used Top2 drugs, such as anthracyclines and etoposide, are poisons which target the active site during the catalytic cycle of the enzyme. The active sites of Top2A and Top2B are very similar, which makes it hard for one to be targeted without hitting the other, though some progress has been made on this front experimentally [20,21,22]. One consequence of this is that there are some severe adverse events associated with Top2 poisons, such as treatment-induced leukemia with etoposide and cardiotoxicity with anthracyclines like doxorubicin [11,19,23,24]. There is evidence that these adverse events are mediated by Top2B [24,25,26,27,28]. Therefore, there is interest in designing specific inhibitors of Top2A, since this isoform is very active in dividing cells (like cancer cells) and is reduced in expression in many differentiated tissues [13,14].

One challenge of designing selective inhibitors of Top2A is that Top2A and Top2B share a high degree of sequence identity through the ATPase and cleavage/ligation domain of the protein: ~81% identity (overall 69% identity for whole sequence). As depicted in Figure 1, the structure of Top2 includes an N-terminal ATPase domain followed by a transducer domain that enables communication between the ATPase domain and the core of the protein [29,30,31,32]. After the transducer domain, there is the TOPRIM domain, which is a metal-binding domain found in topoisomerases and primases [33,34]. The TOPRIM domain is followed by the active site and DNA-binding regions that coordinate with the TOPRIM domain during DNA cleavage and ligation [10]. The next portion is referred to as the C-terminal gate (or C-Gate), which is involved in releasing transported DNA segments from the enzyme [35]. A long α-helix leading up the side of the C-Gate leads to a large intrinsically disordered region (IDR) comprising ~400 amino acids known as the C-terminal domain [36,37,38,39,40,41,42,43,44,45]. This region shares only ~42% identity between Top2A and Top2B and has no stable secondary structure, as is predicted by the Alphafold structure of TOP2A (AF-P11388) [4]. The CTD is important in localization, substrate selection, and regulating the activity of Top2 [37,38,39,44,45,46,47]. The CTD appears to interact with other proteins, including histone 3A [41,43,48]. In addition, the CTD contributes to regulating the isoform specific localization and functions of Top2A and Top2B [37,38,39,40,49,50].

Recent studies have demonstrated that the IDR of the CTD in eukaryotic Top2 (including *S. cerevisiae* Top2 and human Top2A and Top2B) is involved in liquid–liquid phase separation and can form phase condensates with Top2 and DNA [45]. Gene expression and chromatin structure modulation appear to involve phase separation interactions [51,52,53,54]. Consistent with previous studies of the Top2A CTD, the ability to form phase condensates is supportive of a role for the CTD of Top2 in complex interactions that may regulate the biochemical function of Top2 [42,43,44].

Over the last several years, we have aimed to characterize various regions of the CTD and understand the roles of the CTD in biochemical function [43,44]. Using a series of mutants, we analyzed function and identified regions that influence catalytic activity [44,55]. In addition, we employed a bioinformatic tool called PSICalc to analyze the sequence and identify interdependencies in the sequence and develop additional mutants based upon these data [6].

In the present study, we report on the analysis of a previously published Top2A-specific multiple-sequence alignment (MSA) with 105 species [56]. We have examined the MSA using an updated version of PSICalc, and we have identified various significant clusters within the protein and have analyzed them with a focus on interdomain interdependencies. Our data will highlight clusters between separate domains and within the CTD. We compare these results with an analysis of a 125 species Top2B dataset from the same source [56]. Further, we bring key observations together into a model for how PSICalc can be used for protein analysis and what can be learned from this tool about Top2A and Top2B.

## 2. Results

### 2.1. Improvements Made to the PSICalc Software Tool and Shannon Entropy Filtering

**Figure 2:**
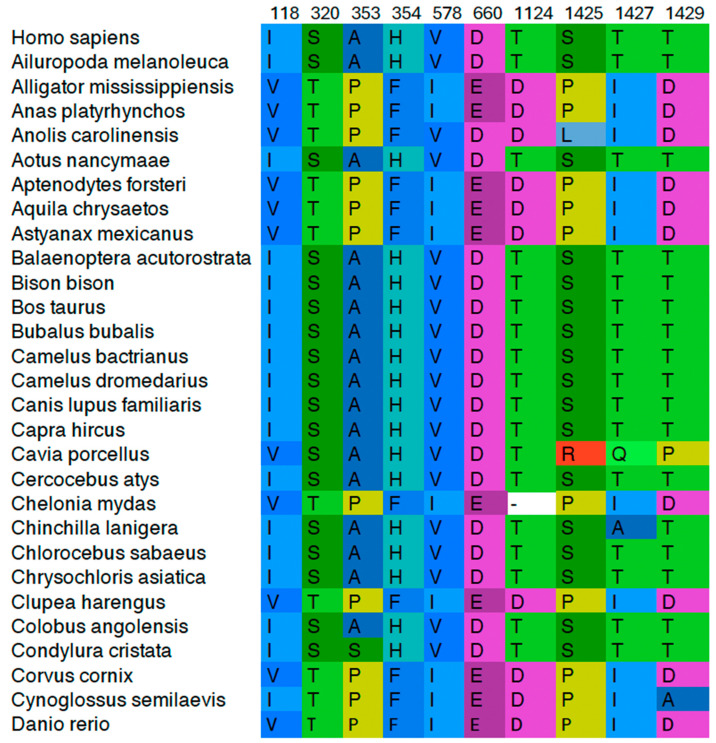
Example cluster from PSICalc analysis with color-coded amino acids. Sites are listed across the top numbered according to human Top2A positions. Species are listed on the left. Cluster truncated for display purposes. Amino acids are color-coded to highlight the interdependencies. Full cluster data available in Supplementary Materials (Data S2).

PSICalc utilizes a derivation of k-modes clustering where Normalized Mutual Information is the metric/distance measure used to compare amino acid changes in a column of an MSA with other columns of the MSA and to discover patterns between columns of the MSA (see Figure 2 for color-coded example). The algorithm discovers relationships between pairs of columns (where each column represents an aligned site in the MSA), and then clusters additional sites making third-, fourth-, fifth-, and higher-order clusters [6]. This pattern discovery approach does not require structural information, which allows it to be employed on both structured and disordered regions. As such, this tool can be applied to the CTD of Top2A and other proteins with IDRs. While the clusters may imply proximal interactions between amino acid positions, these may also imply long-distance interdependencies that may not be obvious based upon structural information. 

In analyzing data from the previous version of PSICalc, it was recognized that some clusters identified in the analysis included one or two high-Shannon-entropy MSA columns (i.e., multiple amino acids found in the column) grouping with clusters of low-Shannon-entropy columns (little change in amino acids within a column) [6]. Examples of false positive clusters can be found in the Supplementary Materials: Data S1, Anomalous Clusters. A very low-Shannon-entropy site (represented by a column in the MSA with little if any variation) may not have sufficient variation to give a statistically reliable association with another site with a much higher degree of variation. For example, PSICalc found clusters between sites 62, 63, 73, and 1317 and between 43, 44, 45, and 1463 (Supplementary Data S1). Upon further inspection, sites 62/63/73 and 43/44/45 show very little variation, while 1317 and 1463 are highly variable.

It was determined that columns with a Shannon entropy (hereafter termed “entropy”) close to 0 (i.e., columns with little variation in amino acids throughout the MSA) were causing a potential issue in the clustering algorithm. Since these columns were nearly invariant (e.g., varying ~1–2 species across the MSA), their significance is recognized in the overall protein structure. In other words, amino acid positions that are shared across all or nearly all species in the alignment are clearly critical to the protein. However, they offer little additional information regarding interdependencies within the protein since there are no clear patterns of association within the MSA. Therefore, we added a feature to the PSICalc software tool (version 0.5.1 and newer) to be able to filter out low-entropy columns using a sliding scale from 0 to 0.25 where the values are a measure of entropy, based upon the calculation used in the software (see Supplementary Materials file for entropy calculation). The calculation ignores gaps/insertions in sequence data and calculates based upon actual amino acid variations. In addition, version 0.5.1 of the software now outputs the full column data for amino acids in each column of a cluster for pairwise up to 10th-order clusters (Figure 2 and full data in Supplementary Materials: Data S2). We re-analyzed the Top2 MSA dataset from our previous paper and present clusters for selected positions in the Supplementary Materials (Data S1, Updated Clusters). 

A previous work by Moreira and colleagues examining Top2A and Top2B generated a dataset of 105 sequences for Top2A that we analyzed using an updated version of PSICalc (0.5.1) with entropy filtering [6,56]. We rearranged the sequences to place human Top2A as the first sequence and used this sequence for generating the position numbers for clusters from PSICalc. Within PSICalc, we adjusted the percentage of non-insertion data until the sequence length matched the length of human Top2A (1531 amino acids). The data were run with a spread of 1, which enables the comparison of each position. In addition, the entropy cutoff was set to 0.1, which removed positions that changed only once or not at all throughout this MSA. Full dataset output is available in the Supplementary Information (Data S2) along with the MSA used in the analysis (MSA File). As seen in the sample shown in Figure 2, PSICalc clusters positions from across the protein by identifying the patterns of amino acids in a given column compared with other columns.

**Figure 3:**
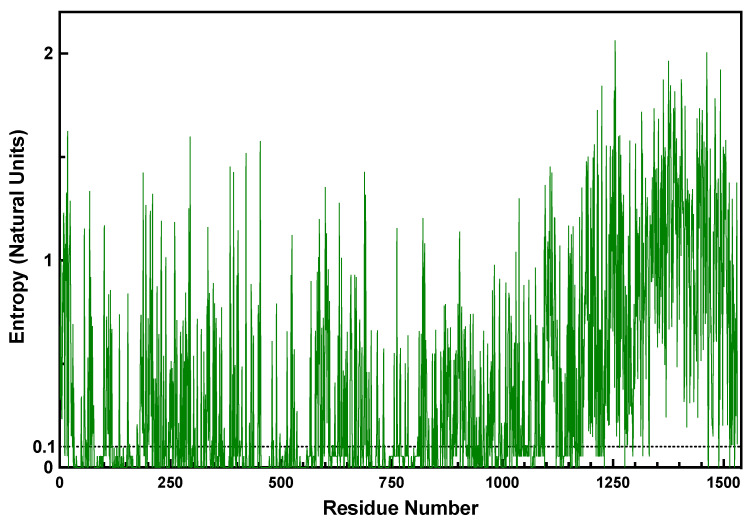
Shannon entropy value (natural units) for each amino acid position in the Top2A dataset. The cutoff value of 0.1 is denoted by the dotted line. Positions below this value were not clustered.

The addition of the entropy cutoff removed a significant number of columns from the dataset under study. Over 700 columns (732 out of 1531 in Top2A) were found to have an entropy score at or below 0.1 and were removed (see Supporting Information for output file). The remaining positions were clustered. As seen in Figure 3, mapping the entropy score of each amino acid position across the protein demonstrates that the most variable region of the protein is the intrinsically disordered CTD (see Supplementary Materials File for Shannon entropy calculation and Figure S1 for entropy distribution). For example, of the 732 columns with low entropy, only 22 were found to be between 1175 and 1531. Thus, only 6.2% (22/356) of the columns in the CTD are low-entropy compared to 60.3% (709/1175) of the N-terminal portion and 47.8% of the whole (732/1531). Interestingly, a review of the positions that were below the entropy cutoff demonstrates that domains involved with the catalytic cycle directly, such as the ATPase, TOPRIM, active-site, and structured DNA-binding domains, are highly invariant among the 105 species examined.

### 2.2. PSICalc Identifies Complex Interdomain Clusters

Upon analysis of the Top2A data, complex clusters spanning multiple domains were identified and examined. As seen in Figure 2, a portion of a 10th-order cluster is shown with positions representing multiple domains (see Supplementary Materials: Data S2 for full output file). Three clusters were selected from the 10th-order cluster set, as outlined in Table 1.

**Figure 4:**
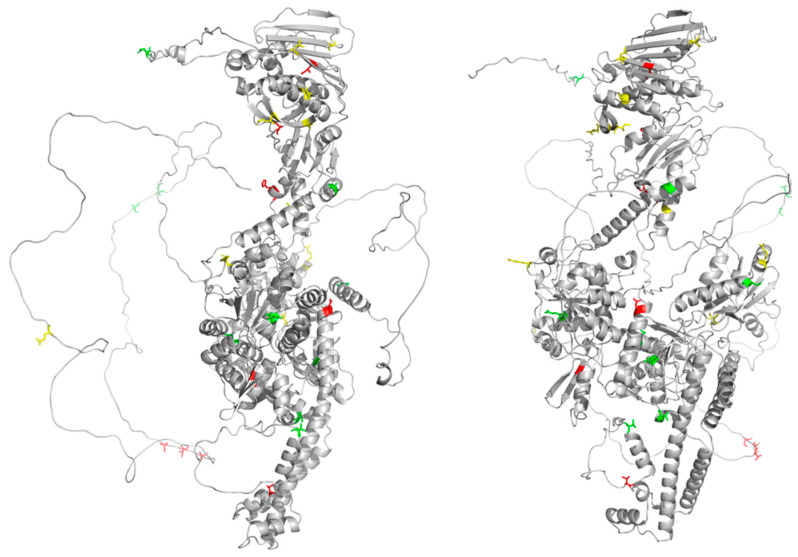
Mapping of 10th-order clusters onto Alphafold structure of Top2A monomer. Three 10th-order clusters are highlighted in red, green, and yellow, corresponding to the colors in Table 1 mapped onto a monomer of Top2A with the intrinsically disordered region shown. Views are rotated 90 degrees relative to each other. Structure from AlphaFold: AF-P11388-F1. Image generated using Pymol 2.5.2.

As seen in Table 1, a strong interdependency is found among clusters of residues spread across the protein. In each case, positions from the N-terminus, core domains, and C-terminus cluster together (Figure 4). The strength of these interdependency relationships is quantified by the statistical redundancy mode (SRMode) value (max value of 0.2 for a 10th-order cluster), which has been defined previously [6]. Visual examination of the columns also shows the interdependence among the amino acid positions (Figure 2; Data S2). It is worth noting that every cluster examined at the 10th-order level included positions from across the domains of the protein (see Data S2). 

Moreira and colleagues also identified six residues in Top2A that influence the activity of topoisomerase II poisons and that differ from the corresponding positions in Top2B: R450, K480, M762, S763, V908, and I909 [56]. Interestingly, each of these positions has a lower pairwise identity within Chordata than the other 21 positions that were examined. They proposed that these sites could be exploited in the development of selective inhibitors. Most of these sites are found in the clustering data with PSICalc. R450 forms a strong fourth-order cluster (240, 338, 450, 1479). Other positions, including M762 (strong pairwise with 637), S763 (as a large cluster), V908 (874, 908, 1060, 1089, 1111, and 1320), and I909 (strong pairwise with 891), also form clusters. These interdependencies may be useful in the design of the next generation of Top2A poisons. 

### 2.3. PSICalc Identifies C-Terminal Domain Clusters

While clusters at the 10th order represented interdomain groupings, smaller clusters that represented groupings within specific domains were also identified. Therefore, clusters that were exclusive to the C-terminal domain were examined. Table 2 contains the top CTD clusters of the third, fourth, and fifth orders from the analysis. At least one cluster represents sites that are adjacent: 1394, 1397, 1399. The remaining clusters are represented by sites spread across the CTD.

Included in several clusters in Table 2 are amino acid positions that were either mutated or are adjacent to positions that have previously been mutated to analyze the CTD of Top2A [44,55]. Some of the mutations had significant impact on catalytic activity, which likely resulted from altered binding/association properties. While the PSICalc clusters do not directly match up with the positions in the mutants, these previous results at least serve as a basis to consider the biochemical effect of changes to the regions under consideration.

### 2.4. Shannon Entropy Filtering Identifies Invariant Sites in the CTD

**Figure 5:**
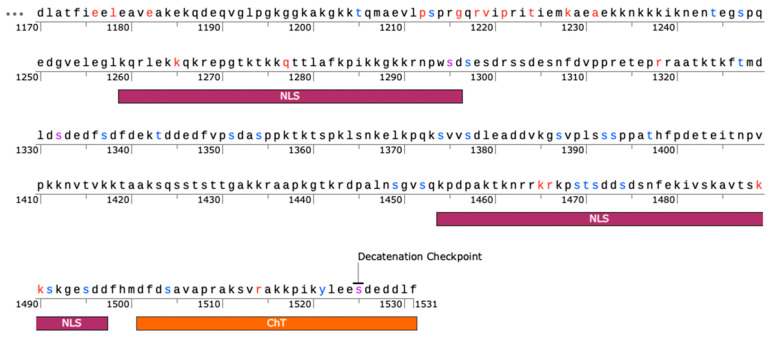
Mapping sites in the C-terminal domain. Low-Shannon-entropy sites are shown in red. Sites that are known to be phosphorylated in association with mitosis are in blue. Purple sites indicate low-entropy sites that are also known phosphorylation sites. Nuclear localization sequences (NLS) and Chromatin Tether (ChT) domains are indicated. Phosphorylation data from Phosphosite Plus. Figure generated using SnapGene version 7.2 (https://www.snapgene.com/, accessed on 22 March 2024).

While the PSICalc algorithm identifies interdependencies among amino acid positions based upon the patterns of intrinsically linked variation, mutual information, it is also recognized that invariant positions have a critical role in protein structure and/or function. Interestingly, while the CTD is the most variable domain, there are 22 sites in the CTD that were filtered out due to low entropy scores (below 0.1 as calculated by PSICalc), which suggests these sites may have critical roles that are shared across various species (Figure 5, Table 3). 

The 22 invariant sites are spread across the CTD, but most invariant sites are concentrated in range of 1175-1295 (Figure 5). The latter portion of this range includes a nuclear localization sequence (NLS) from 1259 to 1296 [48,57]. Additionally, several sites are within the second NLS (1454-1497) [48,57]. Further, K1228 has evidence of being Sumoylated, while S1295, S1332, and S1525 are phosphorylation sites [58]. Phosphorylation of S1525 is associated with the decatenation checkpoint [47].

**Table 3:** Low-Shannon-entropy CTD positions.

Position in Top2A	Amino Acid	Notes	Source
1176	E		
1178	L		
1182	E		
1212	P		
1216	G		
1218	R		
1219	V		
1221	P		
1224	T		
1228	K	SUMO Site	[59]
1231	A		
1265	K	NLS (1259-1296)	[48,57]
1277	Q	NLS (1259-1296)	[48,57]
1295	S	PO_4_ Site; Mitosis-Associated (PlK-1); NLS (1259-1296)	[48,57,59]
1318	R		
1332	S	PO_4_ Site; Mitosis-Associated (PlK-1)	[59]
1465	K	NLS (1454-1497)	[48,57]
1466	R	NLS (1454-1497)	[48,57]
1489	K	NLS (1454-1497)	[48,57]
1490	K	NLS (1454-1497)	[48,57]
1514	R		
1525	S	PO_4_ Site; Decatenation checkpoint	[47,59,60]

### 2.5. Low-Shannon-Entropy Positions Correlate with Charged Positions

**Figure 6:**
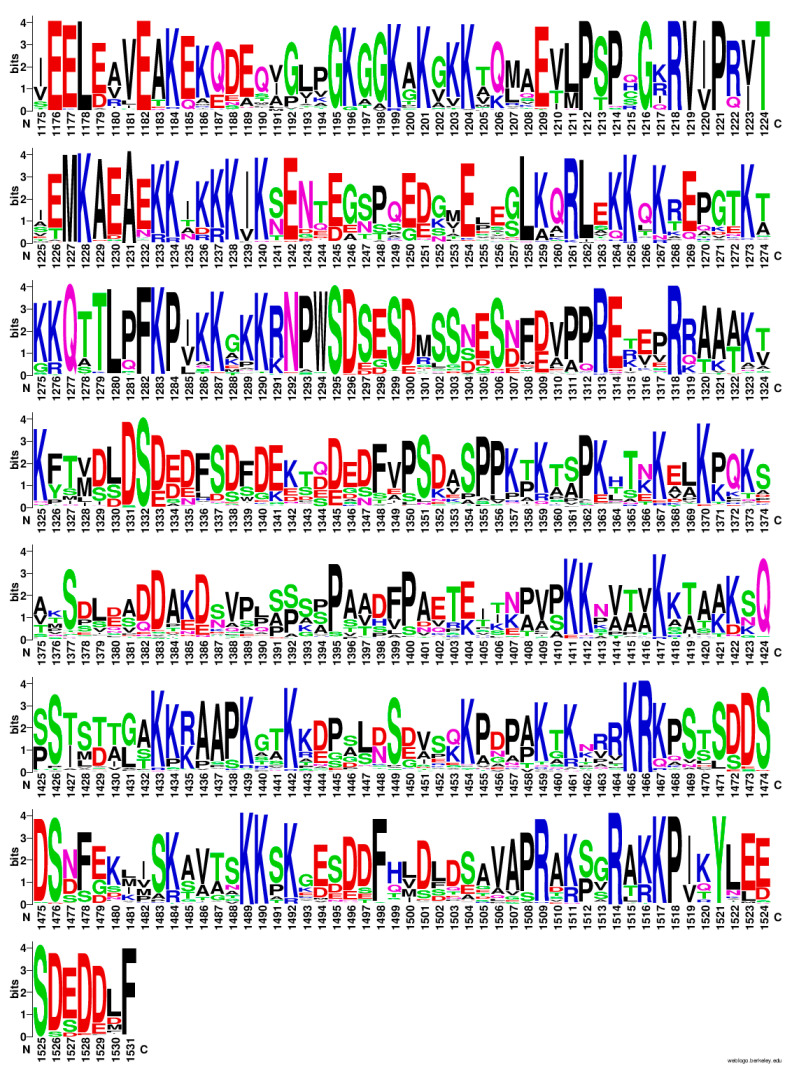
Frequency plot of amino acids in the CTD from the 105 species Top2A alignment. Taller letters indicate the frequency of the amino acid at a given position. Figure generated using WebLogo.

As seen in Table 3 and Figure 5, many of the sites that do not change represent charged sites, especially Arg and Lys. Interestingly, an analysis of the frequency of amino acids at each site in the CTD across the 105 species in the alignment displays some key themes and regions through the CTD. As seen in Figure 6, there is a high proportion of charged residues, especially Lys, Arg, Asp, and Glu, throughout the CTD, and many of these positions are highly invariant, as seen in the sequence logo. In the sequence logo, taller letters indicate the position is less variable within the MSA, which implies higher information content (bits) compared to sites that are more variable. In addition, these residues appear to come in an alternating pattern (positive–negative–positive–negative), especially closer to the end of the sequence. 

From an examination of Figure 6, the 0.1 value for the entropy score cutoff did not catch all the low-entropy positions in the CTD. Positions such as M1227, F1282, K1283, and R1313 are marginally above the threshold but are still mostly invariant. Adjusting the value to 0.11 or higher allows these sites to be removed from the clustering. Re-running that analysis at 0.11 (which included positions that change twice across the MSA such as 1517, 1518) provides a very similar set of clusters, indicating that these sites were not affecting the bulk of the clustering process (See Supplementary Materials: Data S3). 

### 2.6. Analysis of Top2B Dataset Reveals Patterns of Interdependency

**Figure 7:**
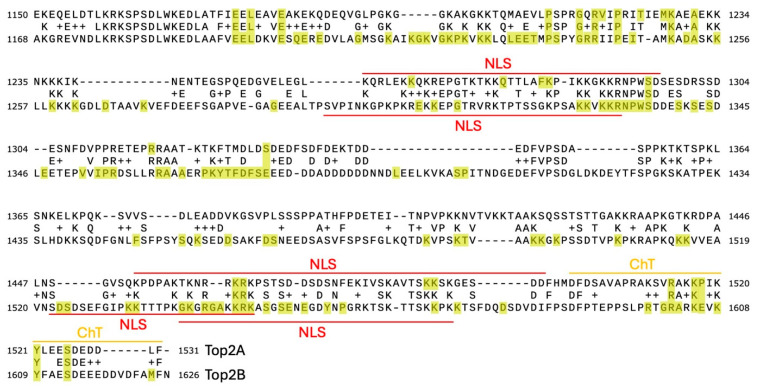
Top2A and Top2B CTD alignment. Human Top2A and Top2B sequences are shown. Highlighted in yellow are low-entropy sites (0.11 cutoff value) for both MSAs. Nuclear localization sequences (NLSs) are in denoted by the red line and the Chromatin Tether (ChT) domain is denoted by an orange line.

As a further step, we also analyzed a 125-species MSA of Top2B, also from Moreira et al. [56]. Top2B has 1626 amino acids, of which 1,012 sites were considered low-entropy, with a 0.11 cutoff, which included over 100 sites in the CTD (See Supplementary Materials: Data S4, Figures S2 and S3). Similar to Top2A, multiple interdomain clusters of 10 positions with high SRmode values are found in the Top2B dataset, including positions from the N-terminal, core, and C-terminal domains (see Supplementary Materials Data S4). In Figure 7, we plot an alignment between the CTD of Top2A and Top2B and annotate low-entropy sites in both enzymes (at a 0.11 cutoff). As seen in Figure 7, many low-entropy sites in Top2A have corresponding low-entropy sites in Top2B. There are some notable exceptions, such as the region between 1259 and 1284 in Top2A (corresponding to one of the two NLSs in Top2A). Several patches of residues are invariant in the Top2B CTD. While some of these regions correlate with the NLSs in Top2B, other regions are relatively unexplored other than identification of post-translational modifications [43,48,57,59]. 

**Figure 8:**
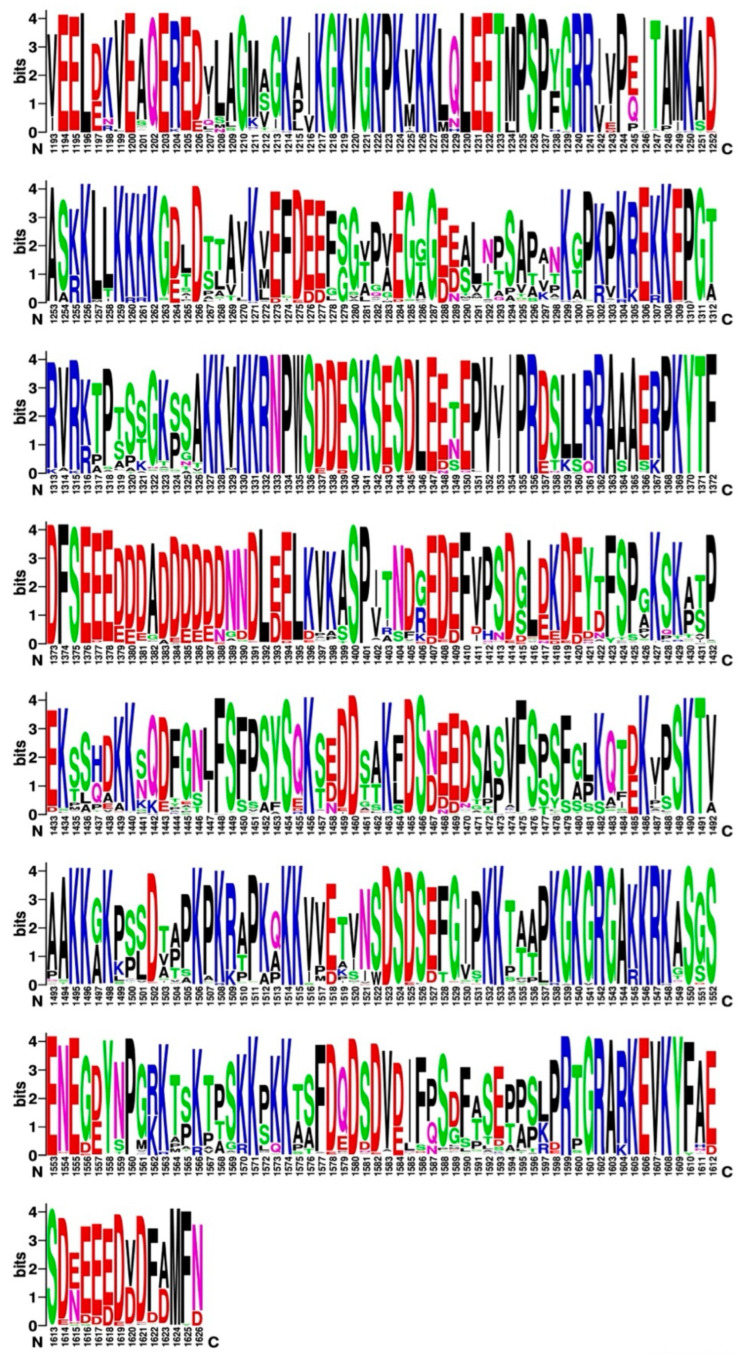
Frequency plot of amino acids in the CTD from the 125 species alignment of Top2B. Taller letters indicate the frequency of the amino acid at a given position. Figure generated using WebLogo.

As seen in Figure 8, the logo diagram for the CTD of Top2B reflects the lower variability of this region. While a similar pattern of positive and negative charges can be seen, there are some noticeable differences in the pattern. One region of note from 1373 to 1394 has a very high proportion of negative charges. While Top2A has some corresponding acidic residues, this region does not completely correlate between the two. Additional studies will be required to explore the role of these regions in Top2A and Top2B. It is possible that these sequences could represent interaction domains for other proteins, as is the case for the region of 1506-1512 in Top2B and 1432-1441 in Top2A, which appear to interact with phospholipid scramblase 1 (PLSCR1) [61].

## 3. Materials and Methods

### 3.1. Top2A and Top2B MSA Datasets

An MSA of Top2A from 105 organisms all from phylum Chordata was obtained from the authors of Moreira et al. [56]. The dataset was modified to place *Homo sapiens* Top2A at the top of the MSA before running the sequences through the PSICalc algorithm. This version was also truncated to remove any gaps in the human Top2A sequence. Full MSA is available as a CSV file in Supplementary Materials. The same approach was also used to prepare the Top2B MSA with 125 sequences from phylum Chordata. These data are also provided in the Supplementary Materials. 

### 3.2. Data Analysis

Interdependency data were collected utilizing PSICalc version 0.5.1. Blue Book for Mac OS available on Github (https://github.com/jdeweeselab/psicalc-package, accessed on 22 March 2024). The data were run by selecting “first row mapping” and setting the percentage of non-insertion data set to 56%. A spread of 1 was selected to compare each column with each other column. The Shannon entropy threshold was set at 0.1 (Supplementary Materials: Data S2) and an additional run was completed at 0.11 (Supplementary Materials: Data S3) for comparison. The analysis in this paper focuses on the dataset from the 0.1 entropy threshold run except where indicated. Top2B was run with a spread of 1, 14% non-insertion threshold and an entropy threshold of 0.11. Data were output as Excel files available in the Supplementary Materials. Structure images were generated using Pymol 2.5.2 from crystallographic and Alpha-Fold structures. Entropy values isolated from PSICalc were plotted using Graphpad Prism 10. The amino acid frequency logo figures were generated using Weblogo (https://weblogo.berkeley.edu/, accessed on 28 February and 26 April 2024). 

## 4. Discussion

In this present study, we used an updated PSICalc clustering algorithm to analyze a dataset of Top2A from 105 species originally published by Moreira et al. [56]. The original PSICalc algorithm errantly clustered one or two high-entropy columns with nearly invariant (low-entropy) columns [Supplementary Materials Data S1]. As a result, spurious clusters were formed. The latest version removes the invariant and nearly invariant clusters using an entropy-filtering approach. In addition, PSICalc now outputs clusters both numerically with SRMode values and as groups of columns representing clustered positions in the MSA numbered according to the first row of the MSA. 

Our results here show that even with the removal of the low-entropy positions, PSICalc still discovers clusters within and among various domains of the protein. Interestingly, we identified several large clusters with moderately strong SRMode values where positions from the N-terminus, core domain, and C-terminus of Top2A are all within the cluster. These groupings imply long-range interdependencies within the protein. While the biochemical details of such interdependencies have not been worked out, the results suggest that interdomain relationships may play a key role in large, multidomain proteins like Top2A. Interdomain interactions have been explored on some level but many details remain to be examined [62].

Further, CTD-specific clusters were also identified and compared with previous results of biochemical experiments characterizing CTD mutants (Table 2). Strikingly, many of the identified interdependent clusters either included one or more mutated position or were adjacent to the mutated positions in our previous study. These mutations were selected by identifying groupings of Ser and Thr residues and mutating them as a group to determine whether those mutations impacted biochemical activity [44]. As noted in Table 2, some of the mutations either increased or decreased plasmid DNA relaxation activity, which may imply that these regions influence substrate selection, substrate binding, stability of the enzyme/DNA interaction, and/or other aspects of the catalytic cycle [44,55]. 

Entropy filtering removed invariant and nearly invariant sites, which are certainly recognized as being critical. Given that almost half of Top2A is essentially invariant across 105 species, it appears that many positions in this enzyme are fixed. This includes 22 positions in the C-terminal domain that were identified by entropy filtering. Interestingly, these are primarily found within one region (~1176–1295). Most of the positions are either charged or polar and several are found within the NLSs. A few are associated with known phosphorylation sites. S1295, S1332, and S1525 are all known to be phosphorylated in a cell cycle-dependent manner and all appear to impact catalytic activity [44,47,55,60,63]. Interestingly, each of these sites were mutated in our previous study looking for roles of clusters of CTD residues [44,55]. While the regions including S1295 and S1332 appeared to decrease relaxation when mutated, no effect on relaxation was observed in the region including S1525, but there was a slight increase in DNA cleavage levels, especially with etoposide [44].

Finally, there appears to be a clear pattern in the CTD of alternating charged residues between Asp/Glu and Arg/Lys (Figure 6). Importantly, the charged residues appear to be sites that are often less variant than positions around these sites. This implies that the charges are likely involved in the function of the CTD. While some charged positions may be explained as being a part of an NLS, other positions could be involved in DNA and/or protein interactions. McClendon et al. proposed that patches of positively charged amino acids in the CTD are potentially involved in the recognition of substrate topology [38]. Deletion of one or more of these patches appears to eliminate the ability of Top2A to differentiate between positive and negative supercoils [38]. Vanden Broeck et al. found that a small flexible “linker” region of the Top2A CTD was visible by cryo-electron microscopy (residues 1191-1217) [62]. Of note, this region includes a series of positively charged amino acids and appears to interact with the Gate-segment of DNA [62]. Our data are consistent with their findings and indicate that this region includes relatively invariant positive charges along with some other critical positions. 

Additional evidence regarding the role of the charged patches in the CTD comes from recent work by Jeong et al., which demonstrates that phase condensation of scTop2, Top2A, and Top2B is sensitive to salt concentration [45]. Increasing concentrations of salt (from 150 up to 400 mM potassium acetate) disrupted the phase condensates formed by Top2 in the presence or absence of DNA [45]. The ionic disruption of these interactions appears to indicate that charges in the CTD are likely important in the interactions between Top2 and potential binding partners, including DNA and proteins. Additionally, the alteration of activity by phosphorylation and other modifications is supportive of this role as well [42,43,58,60].

Considering the above data, various strategies could be employed to identify potential sites for targeting Top2A in a selective manner. One possible strategy is to develop a way to target the ionic interactions between the CTD and various targets. This will require the identification of specific binding partners that influence the activity of Top2 in the nucleus, and then characterize the binding modalities to exploit features that could lead to altered enzyme activity. 

Another strategy for identifying a region of interest is to compare the amino acid positions involved in CTD-only clusters and determine whether those positions are also found in Top2B. Of the CTD-only clusters in Table 2, those with the highest SRMode values include unique amino acid residues as well as those that are identical to analogous positions in Top2B (e.g., see Top2A/Top2B alignment in Supplementary Materials Figure S2 and Table S1). For instance, the top fifth-order cluster includes two positions that are identical in Top2B (D1304/D1345 and D1344/D1387, where the positions are denoted Top2A/Top2B), one position that is similar (Q1217/R1240), and two positions that are different (L1364/K1434 and V1482/T1564). Another fifth-order cluster shows a similar pattern where E1189/V1207, E1232/S1254, and V1513/G1601 differ while T1272/T1312 and A1321/A1364 are identical. Of note, this latter cluster includes positions near the invariant region of Top2A. Based upon our analysis of Top2B most of these positions are variable with the exception of R1240 and G1601.

The Top2B MSA also displays a striking degree of uniformity across 125 species of chordates. Further, the clustering within Top2B reveals interdomain clusters (i.e., between the N-terminal, Core, and C-terminal domains) is typical for this enzyme. The clusters may help clarify interrelationships and coordination between these domains. 

## 5. Conclusions

Using an updated PSICalc algorithm with Shannon entropy filtering combined with clustering outputs allowed for rapid identification of significant clusters through both SRMode value ranking and visual inspection of the clustered sites across the MSA. Analysis of Top2A and Top2B MSA demonstrated that nearly half of Top2A and around two thirds of Top2B is highly constrained across chordate species represented in the alignments. Further, clusters were identified that spread across the domains of Top2A and Top2B. Biochemical and other analyses are needed to determine the significance of such interdomain clusters. It is possible that some of these clusters represent control and communication mechanisms. However, the significance of the low-entropy clusters must also be maintained in these analyses. In addition, it will be critical going forward to identify and more fully characterize the nature of interactions of the Top2 CTD with protein-binding partners.

## Figures and Tables

**Table 1:** Examples of interdomain clusters within topoisomerase IIα identified by PSICalc.

Cluster (AA and Position Numbers) ^1^	SRMode	Domain/Fold
I118, S320, A353, H354, V578, D660, T1124, S1425, T1427, T1429	0.164548	118—ATPase domain 320, 353, 354—Transducer 578, 660—Active Site 1124—C-Gate 1425, 1427, 1429—CTD
D23, A401, Q637, M762, T825, C1008, V1097, I1225, D1378, A1381	0.147561	23—ATPase domain 401—Transducer 637, 762, 825, 1008—Active Site 1097—C-Gate 1225, 1378, 1381—CTD
I70, E74, L115, S183, V240, V338, R450, V928, R982, E1479	0.153242	70, 74, 115, 183, 240—ATPse 338—Transducer 450—TOPRIM 928, 982—Active Site 1479—CTD

^1^ Color-coding corresponds to sites in Figure 4.

**Table 2:** Top2A CTD clusters with the highest SRMode values.

Cluster ^A^	SRMode ^B^	Previously Mutated ^C^	Biochemical Effect ^C^
**3rd Order**	Max: 0.66		
1189, 1232, 1272	0.514864	T1272A, T1274A, T1278A, T1279A	Inc Relaxation
1244, 1256, 1374	0.471158	S1374G, S1377A	Inc Relaxation
1217, 1364, 1482	0.434758	S1351A, S1354G, T1358A, S1361A, S1365G	Dec Relaxation; Slower Decatenation
1206, 1357, 1404	0.43775	ND	
1247, 1249, 1338	0.408826	T1324A, T1327A, S1332A, S1337A, T1343A	Dec Relaxation
1317, 1358, 1499	0.398492	S1351A, S1354G, T1358A, S1361A, S1365G	Dec Relaxation; Slower Decatenation
1394, 1397, 1399	0.359155	S1387I, S1391G, S1392A, S1393A, T1397G	Inc Relaxation; Weaker binding
1390, 1405, 1456	0.379833	Same as above	
1209, 1214, 1411	0.351671	ND	
1336, 1353, 1413	0.344962	S1351A, S1354G, T1358A, S1361A, S1365G	Dec Relaxation; Slower Decatenation
1172, 1306, 1377	0.277244	S1374G, S1377A	None
1227, 1237, 1303	0.168657	S1295A, S1297A, S1302G, S1303G, S1306G	Dec Relaxation
1273, 1476, 1531	0.390435	S1469A, T1470I, S1471A, S1474A, S1476A	Strong Dec Relaxation
**4th Order**	Max: 0.50		
1190, 1316, 1480, 1512	0.331816	S1469A, T1470I, S1471A, S1474A, S1476A And T1487A, S1488I, S1491A, S1495I	Strong Dec Relaxation Inc Relaxation
1198, 1213, 1236, 1383	0.179402	S1387I, S1391G, S1392A, S1393A, T1397G	Inc Relaxation
1247, 1249, 1338, 1485	0.307347	T1324A, T1327A, S1332A, S1337A, T1343A And T1487A, S1488I, S1491A, S1495I	Dec Relaxation Inc Relaxation
1200, 1227, 1237, 1303	0.117365	S1295A, S1297A, S1302G, S1303G, S1306G	Dec Relaxation
**5th Order**	Max: 0.40		
1217, 1304, 1344, 1364, 1482	0.249352	S1295A, S1297A, S1302G, S1303G, S1306G And S1351A, S1354G, T1358A, S1361A, S1365G	Dec Relaxation Dec Relaxation; Slower Decatenation
1189, 1232, 1272, 1321, 1513	0.259725	T1272A, T1274A, T1278A, T1279A	Inc Relaxation
1334, 1336, 1353, 1366, 1413	0.191835	T1324A, T1327A, S1332A, S1337A, T1343A And S1351A, S1354G, T1358A, S1361A, S1365G	Dec Relaxation Dec Relaxation; Slower Decatenation
1323, 1390, 1405, 1453, 1456	0.205813	T1324A, T1327A, S1332A, S1337A, T1343A And S1449A, S1452A	Dec Relaxation None
1200, 1215, 1227, 1237, 1303	0.087985	S1295A, S1297A, S1302G, S1303G, S1306G	Dec Relaxation

^A^ Clustered amino acid positions are numbered according to human Top2A sequence. ^B^ SRMode values calculated by PSICalc algorithm; maximum SRMode value based upon cluster number is also shown. ^C^ From reference [44].

## Data Availability

The original contributions presented in this study are included in the article/Supplementary Material; further inquiries can be directed to the corresponding author.

## Supplementary Materials

The supporting information can be downloaded at https://www.mdpi.com/article/10.3390/ijms25115674/s1.
