# Supplementary material for: Bioinformatic Analysis of Topoisomerase IIα Reveals Interdomain Interdependencies and Critical C-Terminal Domain Residues
Source: Int J Mol Sci. 2024 May 23;25(11):5674. doi: 10.3390/ijms25115674 (PMC11172036; doi:10.3390/ijms25115674)
Supplement: Supplementary file 1 [file ijms-25-05674-s001.zip › Supplementary Materials TOP2A MS.pdf]

Supplementary Materials for:

Bioinformatic Analysis of Topoisomerase IIα Reveals Interdomain Interdependencies and Critical C-Terminal Domain Residues

Clark E. Endsley, Kori A. Moore, Thomas D. Townsley, Kirk K. Durston, and Joseph E. Deweese

Supplementary Materials:

Data S1: Examples of spurious clusters derived from algorithm without entropy filtering (.xlsx file).

Data S2: PSICalc data output using TOP2A MSA with 105 species set at spread of 1, 0.1 entropy, and 56% non-insertion data (.xlsx file).

Data S3: PSICalc data output using TOP2A MSA with 105 species set at spread of 1, 0.11 entropy, and 56% non-insertion data (.xlsx file).

MSA: TOP2A 105 species MSA with human TOP2A as first sequence (.csv file). Note the MSA has been truncated to omit regions not found in human Top2A.

Figure S1 (in this document): Shannon Entropy Distribution

Figure S2 (in this document): Shannon Entropy Plot for Top2B MSA

Figure S3 (in this document): Top2B MSA Frequency Distribution of Shannon Entropy Values

Table S1 (in this document): Example Clusters Compared between TOP2A and TOP2B CTD.

Shannon Entropy Calculation in PSICalc – Modified from scikit learn version under the BSD-3 Clause License to remove gaps in sequences as noted below. Full code in context available here: https://github.com/jdeweeselab/psicalc-package/blob/main/psicalc/nmi.py#L163-L184

```
def entropy(labels):
    """
    Unlike the sci-kit entropy function, we are modifying it
    to remove gap labels so they do not interfere with the
    entropy result.
    """
    # non-zero labels i.e. no gaps as gaps are always 0 in psicalc
```

```
nz_labels = labels[labels != 0]
if len(nz_labels) == 0:
   return 1.0
labels_u, label_idx = np.unique(nz_labels, return_inverse=True)
if labels_u[0] == 0:
   label_idx = label_idx[label_idx != 0]
pi = np.bincount(label_idx).astype(np.float64)
pi = pi[pi > 0]
if pi.size == 0:
   return 0.0
pi_sum = np.sum(pi)

# log(a / b) should be calculated as log(a) - log(b) for
# possible loss of precision
return -np.sum((pi / pi_sum) * (np.log(pi) - log(pi_sum)))
```

Figure S1: Shannon Entropy Distribution

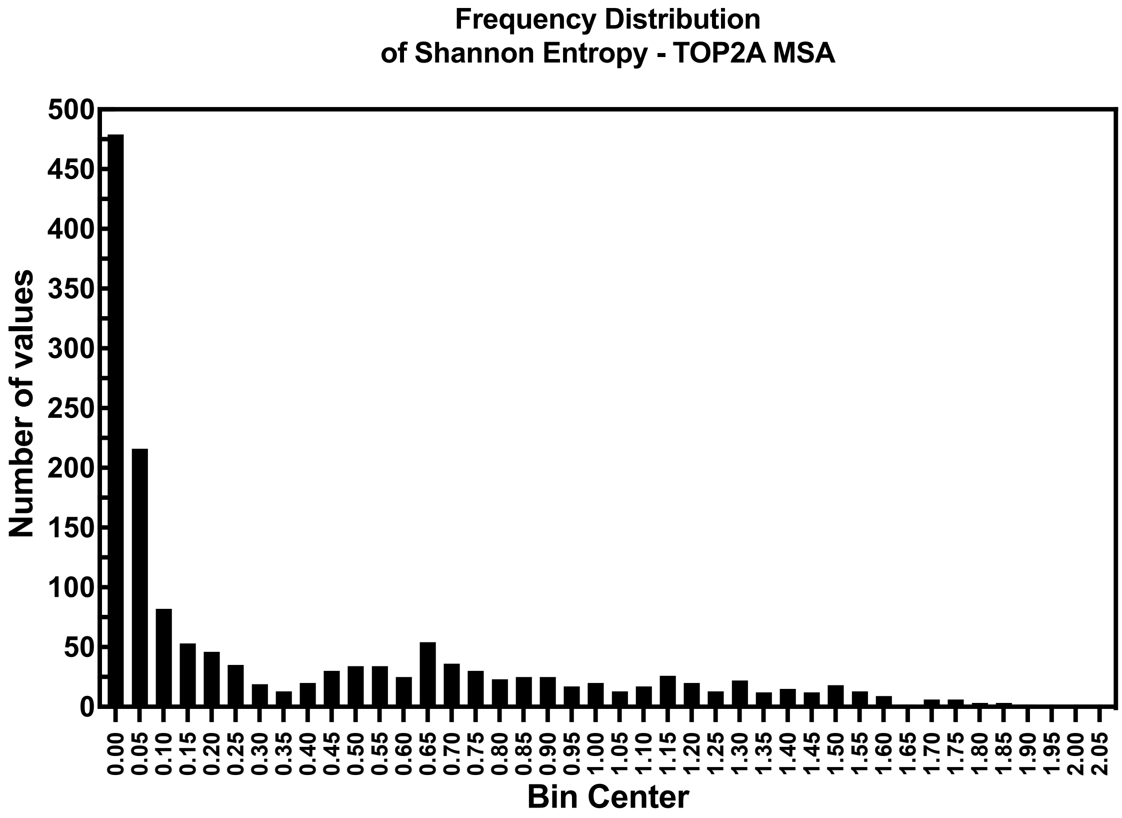

Figure S2: Shannon Entropy Plot for Top2B MSA

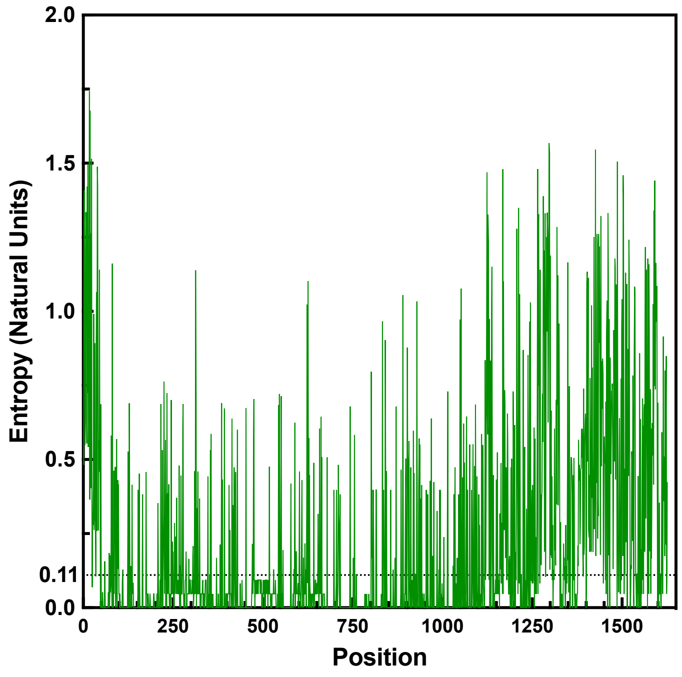

Figure S3: Top2B MSA Frequency Distribution of Shannon Entropy Values

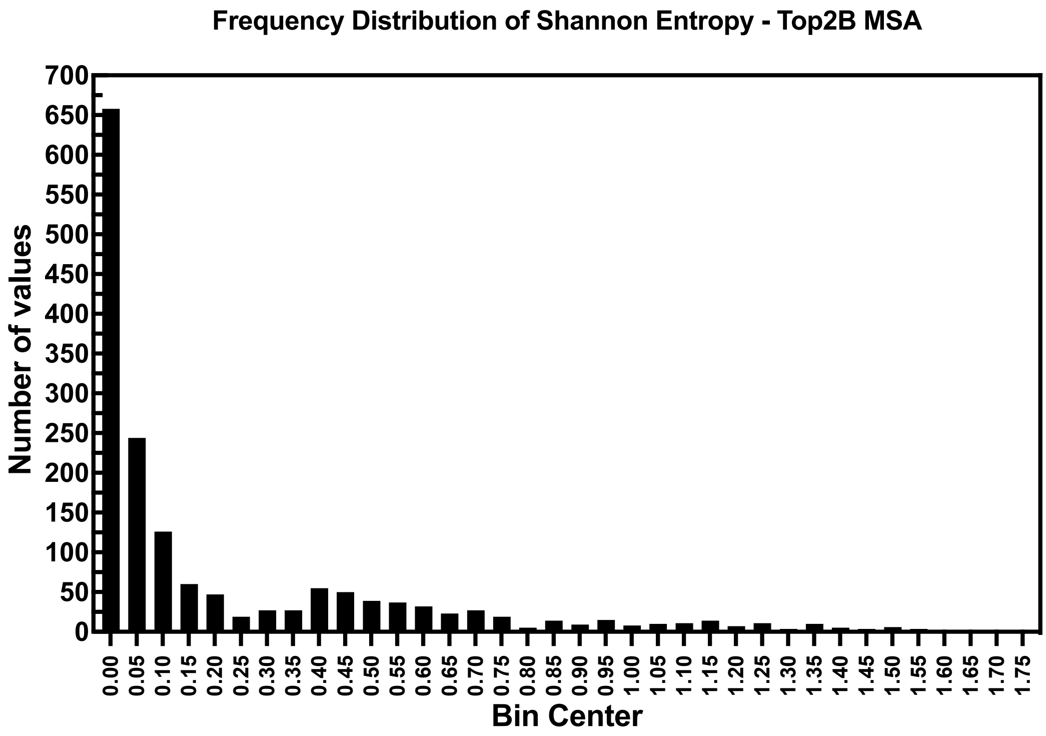

Table S1: Example Clusters Compared between Top2A and Top2B CTD.

Cluster	TOP2A Position	TOP2B Position
1	Q1217	R1240
1	D1304	D1345
1	D1344	D1387
1	L1364	K1434
1	V1482	T1564
2	E1189	V1207
2	E1232	S1254
2	T1272	T1312
2	A1321	A1364
2	V1513	G1601
3	Q1190	L1208
3	E1316	L1359
3	K1480	R1562
3	S1512	T1600

Red denotes differences; blue denotes similarities; black denotes no change. Clusters are from Top2A data and mapped to Top2B using alignment in Figure S1.
